# Use of disposable endoscope for variceal sclerotherapy

**DOI:** 10.1055/a-2215-1100

**Published:** 2023-12-15

**Authors:** Kisako Fujiwara, Takayuki Kondo, Kentaro Fujimoto, Mai Fujie, Naoya Kanogawa, Sadahisa Ogasawara, Naoya Kato

**Affiliations:** 1 Gastroenterology, Graduate School of Medicine, Chiba University, Chiba, Japan; 2 Gastroenterology, Graduate School of Medicine, Chiba University, Chiba, Japan; 3 92154 Department of Clinical Engineering Center, Chiba University Hospital, Chiba, Japan

Diagnostic esophagogastric endoscopy has recently been performed using new sterile single-use disposable endoscopes (Ambu aScope Gastro; Ambu, Ballerup, Denmark) ( Fig. 1 ) due to the potential risk of cross-infection associated with reusable endoscopes 1 2 .

**Fig. 1:**
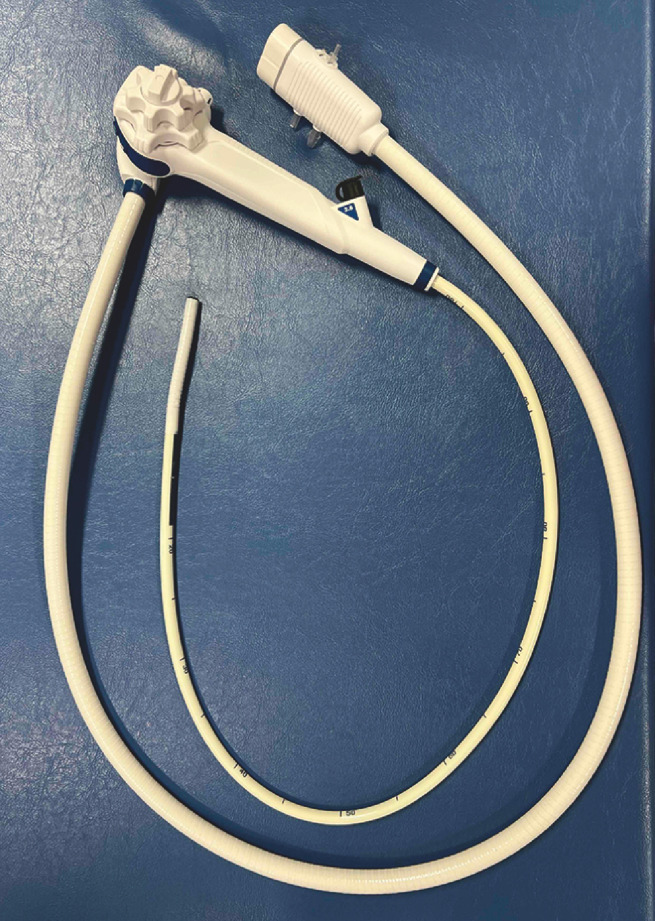
Ambu aScope Gastro.

N-butyl cyanoacrylate (NBCA) is used for emergency endoscopic hemostasis in cases of gastric and ectopic varices 3 4 . However, one of the complications associated with the use of NBCA during sclerotherapy is its adhesion to the endoscope, which causes critical damage to the endoscope and incurs high repair costs. In addition, the puncture site can overlap with the endoscope on the fluoroscopic images during sclerotherapy.

We report two cases in which sclerotherapy was performed using the Ambu aScope Gastro for treating gastric and jejunal varices ( Video 1 ).

Gastric and jejunal variceal sclerotherapy using the Ambu aScope Gastro.Video 1

Case 1 is of a 65-year-old man who was referred for the treatment of gastric varices due to alcoholic cirrhosis. The gastric varices were punctured using a 23-G esophageal varices puncture needle (Sumitomo Bakelite., Tokyo, Japan) ( Fig. 2 ). Under fluoroscopic guidance, 1.6 mL of 75% NBCA (NBCA:lipiodol ratio 3:1) was injected. A small quantity of NBCA adhered to the endoscope after the procedure.

**Fig. 2:**
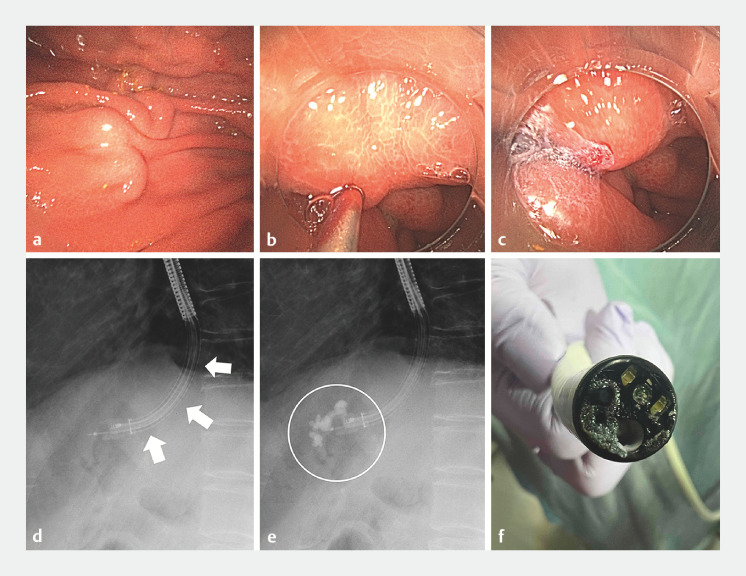
Use of Ambu aScope Gastro for gastric variceal sclerotherapy. **a** Gastric varices. **b** Varices were punctured with a 23-G needle. **c** N-butyl cyanoacrylate (NBCA) administration. **d,e** Radiolucent tip of the endoscope allows easier evaluation of contrast flow (arrow: endoscope). **f** Adherence of NBCA to the scope.

Case 2 is of a 76-year-old man ( Fig. 3 ) who was referred by a surgeon following pancreaticoduodenectomy for an intraductal papillary mucinous adenoma because of suspected bleeding from jejunal varices formed at the site of choledochojejunostomy. The jejunal varices were punctured using a 23-G esophageal varices puncture needle. Under fluoroscopic guidance, 2 mL of mixture of 60% NBCA (NBCA:lipiodol ratio 3:2) was injected. The transparency of the soft tissue scope enabled easy confirmation of the flow and polymerization of the sclerosing agent on fluoroscopic images.

**Fig. 3:**
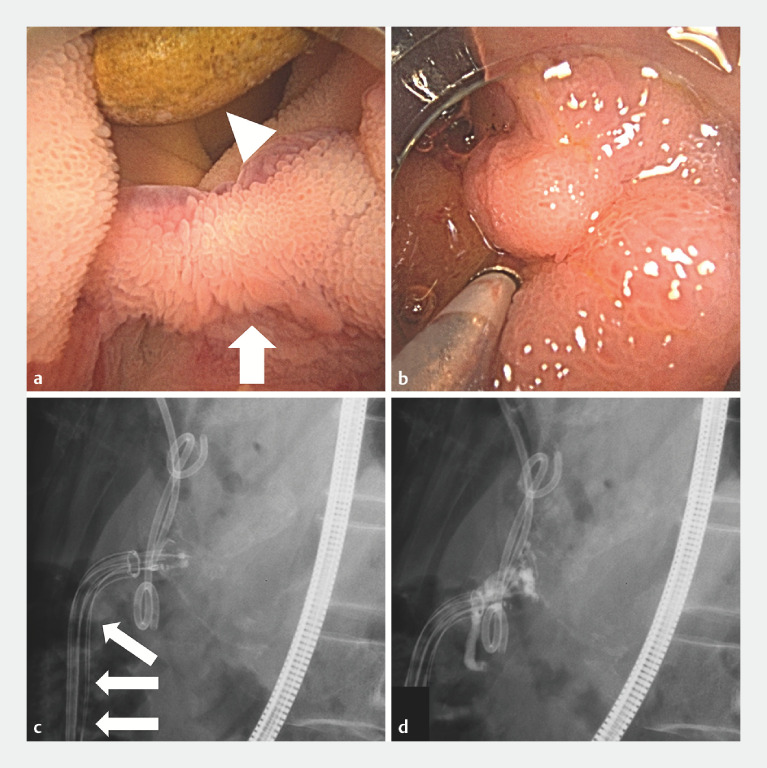
Use of Ambu aScope Gastro for jejunal variceal sclerotherapy. **a** Jejunal varices formed at the site of choledochojejunostomy (arrow). A biliary stent was implanted for biliary strictures (arrowhead). **b** Jejunal varices were punctured with a 23 G needle. **c,d** Radiolucent tip of the endoscope allows easier evaluation of contrast flow (arrow, endoscope).

These cases demonstrate the successful use of sclerotherapy with NBCA using the Ambu aScope Gastro. This single-use disposable endoscope prevents the need for costly endoscope repair following NBCA adhesion and provides excellent visibility on fluoroscopic images.

Endoscopy_UCTN_Code_TTT_1AO_2AD

## Notes

Endoscopy E-Videos https://eref.thieme.de/e-videos
*E-Videos* is an open access online section of the journal *Endoscopy* , reporting on interesting cases and new techniques in gastroenterological endoscopy. All papers include a high-quality video and are published with a Creative Commons CC-BY license. Endoscopy E-Videos qualify for HINARI discounts and waivers and eligibility is automatically checked during the submission process. We grant 100% waivers to articles whose corresponding authors are based in Group A countries and 50% waivers to those who are based in Group B countries as classified by Research4Life (see: https://www.research4life.org/access/eligibility/ ). This section has its own submission website at https://mc.manuscriptcentral.com/e-videos .
